# Comparative Efficacy of an Organic Acid Blend and Bacitracin Methylene Disalicylate as Growth Promoters in Broiler Chickens: Effects on Performance, Gut Histology, and Small Intestinal Milieu

**DOI:** 10.4061/2010/645150

**Published:** 2009-11-30

**Authors:** Saikat Samanta, Sudipto Haldar, Tapan Kumar Ghosh

**Affiliations:** Department of Animal Nutrition, Faculty of Veterinary & Animal Sciences, West Bengal University of Animal & Fishery Sciences, 37 Kshudiram Bose Sarani, Kolkata, West Bengal 700037, India

## Abstract

This study evaluated the efficacy of organic acids as a growth promoter for broiler chickens relative to antibiotic growth promoters (AGPs). Broiler chickens were supplemented with graded doses of an organic acid blend (OAB, 1 g and 2 g/kg diet) and bacitracin methylene disalicylate (BMD, 0.5 g and 1 g/kg diet) for 35 days. Supplementation of OAB improved (*P* < .001) feed conversion ratio (FCR) and increased protein accretion (*P* < .001). Dietary acidification caused pH of the gizzard to decline linearly (*P* < .01) with the dose of supplemental OAB. In the lower intestine, pH remained unaffected by dietary treatments. Unlike BMD, supplemental OAB selectively promoted growth of lactobacilli in the small intestine. Moreover, compared to BMD, OAB tended to maintain the villi in the small intestine at a greater height. Although benefits of exceeding the dose of supplemental organic acids more than 1 g/kg diet are not always conspicuous, based on the live weight and feed conversion data, supplementation of 2 g organic acid per kg diet may be recommended for total replacement of AGPs in broiler diet.

## 1. Introduction

The ban imposed on antibiotic growth promoters (AGPs) in livestock and poultry diets initiated a worldwide search for effective alternatives which would be equally efficacious and have no negative impact on animal welfare and consumer health. Performance of livestock and poultry is closely related to the qualitative and quantitative microbial loads in the alimentary tract of the host animal [1]. The bacterial population in the alimentary tract consists of both pathogenic and beneficial commensals and growth inhibition of the former would facilitate the population of the latter. Owing to the short life span in broilers, the later the beneficial microorganisms colonize in the intestinal tract, the more susceptible the birds become to infection caused by intestinal and environmental pathogens. 

Organic acids such as citric, propionic, fumeric, and formic acids increased gastric proteolysis and improved digestibility of protein and amino acids. Additionally, these acids have been shown to inhibit the growth of intestinal bacteria which compete with the host animal for availability of nutrients and reduced toxic bacterial metabolites such as ammonia and amines. As a result, feed efficiency and growth performance of the host improved as it was reported in poultry [2, 3] and pigs [4]. However, in poultry, performance enhancement by dietary acidification is not as convincing as it is in pigs. Nevertheless, some workers reported encouraging results with fumaric, propionic, sorbic, and tartaric acids [5, 6] and dietary supplementation of formic or propionic acid was reported to reduce the number of enteric pathogens [7] and the incidence of *Salmonella* in carcass [8–10]. 

In this experiment, broiler chickens were supplemented with graded doses of an organic acid blend (OAB) to evaluate the efficacy as a growth promoter compared to AGP and to study the effects of these two dietary supplements on gut morphology, alimentary tract pH, and intestinal microbial counts.

## 2. Materials And Methods

The experimental protocol was approved by the Institutional Animal Ethics Committee. A mixed flock of 200 one-day- old Kasila broiler chicks (developed by GPS Poultry, USA from Hubard parent line) was purchased from a local hatchery. Glucose and electrolyte solutions were offered to the birds upon arrival at the experimental room and ground corn was offered within 12 hours of hatching. Following weighing, 180 chicks were randomly assigned to 5 dietary treatments and 20 chicks were kept separately for a pre-experimental slaughter. Each treatment group consisted of 6 replicates with 6 birds per replicate. The chicks in a replicate were placed in individual pens (0.5 m × 0.5 m) on litter composed of fresh wood shavings. The pens were separated with plastic wire netting and contained plastic feeder and water troughs. Temperature inside the poultry house was maintained through heating elements fitted in the room at 35°C during the first 2 weeks, between 28 and 30°C during the subsequent couple of weeks and at about 25°C in the last week of the study. Continuous light was provided through compressed fluorescent lamps during the first 2 weeks of brooding. In the third week a dark period of one hour duration was introduced in the night and after 21 days the lights were turned off for 4 hours at night to reduce the activity of the birds. Exhaust fans were fitted in the experimental room to ensure adequate ventilation. The birds were vaccinated against New Castle disease (day 7 and 21) and infectious bursal disease (day 14). The starter (days 1–21) and the grower (days 22–35) diets (Table 1) fed to the birds were formulated to meet nutrient requirements [11]. The birds were fed *ad libitum* and a weighed quantity of feed was offered replicate-wise in equal proportions at 08 : 00 and 16 : 00 hours. The total duration of the experiment was 35 days.

The dietary treatments were comprised of feeding the birds with an unsupplemented basal diet (control), supplementation of the basal diet with either 1 g or 2 g of an organic acid blend (OAB) per kg diet (OAB 1 and OAB 2, resp.) and either 0.5 g or 1 g bacitracin methylene disalicylate (BMD, containing 10% active BMD per kg diet (BMD 0.5 and BMD 1 resp.). OAB contained orthophosphoric acid (400 g/kg), formic acid (150 g/kg), propionic acid and calcium propionate (30 g/kg resp.) in a powdered base (Acidbac^TM^, Dex ibèrica, Vila-Seca, Tarragona, Spain). The diets for the designated treatment groups were prepared in lots of 50 kg, and the supplements (OAB and BMD) were weighed and then mixed with 1 kg of respective diets in a small capacity mechanical blender. The mixture thus produced was then added to the remainder of the diet placed in a mechanical blender, and mixed for 30 minutes. The commercially applicable dose rates for both the organic acid blend and the antibiotic growth promoter were applied in this study.

Feed intake was recorded by replicate daily. The total body weight of a replicate was measured at the beginning and at the end of the study and the gain in body weight was calculated replicate-wise. Feed conversion ratio (FCR) was also calculated replicate-wise as a ratio between the total feed intake by a replicate and the total body weight gain of that replicate. Mortality, if any, was recorded and the data were adjusted accordingly. 

As stated above an initial slaughter of 20 chicks was performed on day 0 to determine some pre-experimental carcass traits to be described later and the birds were harvested finally on day 35 by decapitation. The slaughter was performed 2 hours after feeding. The birds were defeathered and eviscerated manually. The feet and heads were removed and the carcass was split longitudinally. The hot carcass was weighted and chilled at 1°C for 48 hours. The chilled carcass was separated into cuts, and the breast, thighs, back, drumsticks, and giblets (gizzard, liver, lungs and heart) were weighed. The cuts were frozen at −20°C for analysis. The frozen cuts from the left side of each carcass were homogenized in a custom made tissue homogenizer. The minced subsamples were repeatedly taken and mixed until a final sample of 500 g was obtained. The final sample was frozen pending analysis of moisture, ash (AOAC 1984), and crude protein (N × 6.25). N was analyzed in an automatic analyzer (Kjeltec Auto 1013 Analyzer, Foss Tecator, Sweden). The samples from the initial slaughter group were also analyzed similarly. Protein and ash accretion was determined by subtracting the total quantity of the a forementioned nutrients in carcass on d 35 from those in the initial slaughter group.

Following sacrificing of the birds on d 35 for carcass traits, the intact gastrointestinal tract from the esophagus to the cecum was separated and placed in sterile Petri dishes after washing with normal saline to remove exterior blood and tissue debris. The gastrointestinal tract from 2 birds in a replicate of a treatment group was cut into segments and the digesta from the crop, proventriculus, gizzard, duodenum and ileum were gently expelled into plastic containers and pooled by replicate. The pH of the pooled sample was determined (Digital pH meter, Model 335, Systronics, Thiland) following dilution to 10 ml with deionized water. The small intestine (from the end point of the gizzard up to the ileo-caecal junction) was dissected from 2 birds of a replicate in each treatment group and the duodenum, jejunum, and ileum were separated. The digesta were taken from each segment in sterile Petri dishes and pooled by replicate. One gram of the pooled digesta was taken segment wise in sterile test tubes and diluted to 10 ml with sterilized deionized water. The contents were then serially diluted and a final dilution of 10^−5^ was used for enumeration of *Escherichia coli*, *Clostridium, Salmonella *total coliforms and *Lactobacillus *spp. For this, 0.1 ml diluted digesta was spread with a sterile platinum loop on the surface of bacteria specific culture media (Hi Touch Flexi Plates, Hi Media Laboratories Ltd., Mumbai, India) and incubated at 37°C for 24 to 48 hours. The colonies were enumerated using a colony counter (manufactured by Optics Technilogy, New Delhi, India) the and the results were expressed as log _10_  Colony Forming Units (CFU)/g digesta.

The starter and the finisher feeds were also analyzed in a similar way for total coliforms, *E. coli*,* Clostridium, Salmonella,* and moulds (*Aspergillus flavus* and *A. parasiticus*). For this, 1 g of finely ground feed sample was mixed with sterilized deionized water and cultures were grown on specific culture media mentioned above following serial dilution. Growth of the microorganisms and the molds was tested after 24 to 48 hours and the colonies were enumerated. 

The duodenum, jejunum, and ileum obtained from each of the birds in a replicate of a treatment group were processed further for estimation of villus height. The segments were cut into small pieces not exceeding 2 mm in length and immersed into alcoholic Bouin's fluid (containing 150 ml 80% ethyl alcohol, 1 g picric acid, and 60 ml formaldehyde with 50  ml glacial acetic acid added immediately before use) for a period of 16 hours. The fixative was then poured off and the tissue was washed thoroughly with 70% alcohol and dehydrated in ascending grades (80%, 90%, and absolute) of alcohol. The tissue segments were then immersed in a solution containing alcohol and cedar wood oil (1 : 1 volume/volume) for 16 hours and then kept in cedar wood oil for 48 hours. The tissue segments were then transferred to a solution containing xylene and paraffin wax at a ratio of 1 : 1 and kept in an incubator at 56 to 58°C for 2 hours with a change in paraffin solution after 1 hour. A quantity of molten paraffin was poured into a small paper block into which the tissue was then transferred and the paraffin was allowed to harden and cut into suitable blocks from which tissue sections were cut at 6 *μ*m with the help of a microtome. The sections were placed on glass slides and kept on a hot plate to get the sections adhered on the slides. The slides were then stained with Delafield's Hematoxyline and Eosin and mounted on Distrene Plasticiser Xylene. The length of the villi was measured with an oculometer under a microscope fitted with a stage micrometer. For each specimen, at least 10 fields were scanned and the mean value for the 10 observations was considered to be a measurable unit for each specimen during subsequent calculations.

The data were analyzed by the general linear model of the SPSS (10.0) and the results were expressed as mean and pooled standard error of mean (SE). The replicates were the experimental units. The control group was used as a common control for both the OAB and the BMD dietary groups. The results were subjected to ANOVA to compare the mean effects of dietary treatments (OAB and BMD) irrespective of the dose levels. Separate analysis was performed employing polynomial contrast to determine the effects of varying dose levels within a dietary treatment. A probability less than 0.05 was considered significant and less than 0.1 was considered trend. 

## 3. Results

There was no difference in chemical analysis results between the starter and finisher diets (Table 2). Total coliforms in the starter and the finisher diets decreased (*P* < .05) due to supplementation of OAB and BMD and the difference between the dietary treatments was not significant in this regard (*P* > .1). *E. coli* and *Clostridium *in both the starter and the finisher diets decreased linearly (*P* < .05) with the dose of the supplemental OAB. When BMD was supplemented, *E. coli* decreased (*P* < .05) compared to the control diet, although in the starter feed the dose of BMD had little effect on dietary *E. coli* count (*P* > .1). However, *E. coli* in the finisher diet and clostridia in both the starter and finisher diets decreased linearly (*P* < .05) with the dose of the supplemental BMD in diet. *Salmonella* test was negative for all the three dietary groups. The growth of *A. flavus* and *A. parasiticus* was either detected sporadically or was not detected at all in most of the cultures. Some mucoid growths were observed in the OAB supplemented diets. Growth of *A. flavus* was higher in the BMD 0.5 than other diets (*P* < .05). *A. fumigatus* was, however, negative in both the OAB and BMD supplemented diets (Table 2). 

Data related to live weight, live weight gain, FCR, gross carcass traits, and chemical composition of meat (Table 3) indicated that live weight increased linearly (*P* < .01) due to supplementation of either OAB or BMD to the diets. Consequently, live weight gain improved in the a forementioned treatment groups (*P* < .01). Cumulative FCR was better (*P* < .001) in the birds supplemented with OAB than those supplemented with the BMD. Among the OAB dietary groups, FCR was better (*P* < .001, linear and quadratic effects) in the OAB 1 group compared to that in the OAB 2 group of birds. Similarly, the BMD 0.5 dietary group had a better FCR than the BMD 1 group of birds (*P* < .05, linear and quadratic effects). No mortality was recorded during the study.

The dose levels of supplemental OAB had no effect on the dressing percentage (*P* > .1). However, dressing percentage improved linearly (*P* < .01) with the dose of supplemental BMD. Irrespective of dose, dressing percentage of the OAB and BMD supplemented dietary groups was similar (*P* > .1). Breast weight increased linearly (*P* < .05) with the dose of OAB and BMD in diet. Dietary supplemental OAB tended to increase thigh weight (*P* < .1). Contrarily, added BMD in diet resulted in a linear increase in weight of the thighs (*P* < .05). Cumulatively, the difference between the OAB and BMD supplemented dietary groups was not significant with regard to the weight of the breast and thighs (*P* > .1). Supplemental OAB had little impact on weight of the drumsticks (*P* > .1). On the other hand, when BMD was supplemented to the diet, the weight of the drumstick increased with the dose (*P* < .001, linear effect). 

Moisture in meat ranged from 72.2% to 73.9% in different experimental groups and the dietary treatments had little effect on meat moisture content (*P* > .1). Irrespective of dose, supplementation of OAB increased meat ash content and total ash accretion more than BMD did (*P* < .001). In the OAB 1 dietary group meat ash content was higher (*P* < .001, linear and quadratic effects) than the control and OAB 2 dietary groups. Supplemental BMD, however, had little effect on meat ash content with little variation being observed between the BMD 0.5 and BMD 1 dietary groups. Supplementation of OAB and BMD did not affect meat protein content (*P* > .1). Meat protein tended to increase (*P* < .1) with the dose of supplemental BMD, although any dose dependent effect with OAB was lacking in this regard. However, protein accretion increased linearly as the dose of supplemental OAB (*P* < .001) or BMD (*P* < .05) increased in diet. Irrespective of the dose levels, protein accretion in the OAB supplemented group of birds was higher than that in the BMD supplemented dietary groups (*P* < .001).

Supplemental OAB and BMD had little effect on pH of the crop (*P* > .1). The pH of the proventriculus also did not reveal marked change when OAB was supplemented to the diet (*P* > .1), although the same tended to decrease with the dose of supplemental BMD in diet (*P* < .1). On the other hand, dietary acidification caused the pH of the gizzard to decline linearly (*P* < .01) with the dose of supplemental OAB. However, supplementation of BMD did not yield any significant effect (*P* > .1) on the pH of the gizzard. Dietary supplementation of OAB or BMD did not affect pH of the duodenum (*P* > .1). In the ileum also the pH remained unaffected by supplementation of OAB (*P* > .1), although in the ileum it tended to be higher (*P* < .1) in the BMD 0.5 group compared to that in the BMD 1 dietary group (Table 4).

The population of *E. coli, Lactobacillus,* and other coliforms in the digesta content of the small intestine is presented in Table 4. Dietary supplemental OAB and BMD did not affect *E. coli* in the small intestine (*P* > .1). Other coliform counts were similar in the OAB and the BMD supplemented dietary groups (*P* > .1). *Lactobacillus* was quadratically higher in the OAB 1 group than the control and the OAB 2 dietary groups (*P* < .001). Addition of BMD in diet had no effect on gut *Lactobacillus* population (*P* > .1). Overall, supplementation of 1 g OAB/kg diet was found to be superior (*P* < .01) in terms of intestinal *Lactobacillus* count to the control and other experimental groups. *Salmonella* and *Clostridium* tests were negative across the dietary treatments.

Supplementation of OAB and BMD had variable effect on the villus height in different segments of the small intestine (Table 4). The general trend indicated that supplementation of organic acids facilitated the growth of the villi compared to the BMD. In the duodenum, villus height increased linearly with the dose of the organic acids (*P* < .05) and the trend was similar in the jejunum (*P* < .05, quadratic effects) and the ileum as well (*P* < .05, linear and quadratic effects). The height of villi in the BMD supplemented dietary group also increased linearly in a dose dependent manner (*P* < .01, linear and quadratic effects). Interestingly, the height of the ileal villi in the OAB 1, BMD 0.5, and BMD 1 dietary groups was lower (*P* < .05) relative to the control group of birds.

## 4. Discussion

Dietary supplementation of organic acid reduced *E. coli* and *Clostridium* more efficiently than BMD. The effect of organic acid in reducing total coliforms was comparable to BMD. It may be noted that despite the dietary samples being positive in *Salmonella* and *Clostridium* the same could not be detected in the alimentary tract of the birds. Perhaps the dietary load of these bacteria was not sufficient enough to induce a clinical infection and the organisms failed to establish themselves in the gut due to competitive antagonism from other commensals present in the gut. The effects of organic acids and BMD on the growth of *Aspergillus* spp. were somewhat inconsistent. Nevertheless, it appeared that addition of organic acids and BMD resulted in complete disappearance of *Aspergillus fumigatus* in the feed samples. 

In the present investigation, the organic acids were found to be superior to BMD in enhancing live performance of the experimental birds probably by their beneficial effects on gut micro flora. Øverland et al. [12] reported that organic acids such as formic acid, elicit bactericidal effects by reducing the intracellular pH of gut micro flora which in turn may improve livestock performance. It is worth mentioning here that *Lactobacillus* is capable of growing at a relatively lower pH and thus is more resistant to changes in gut milieu induced by dietary acidification [13]. In poultry, pathogenic bacteria like *Salmonella* enter the gastrointestinal tract via the crop. The microbial composition and pH of the crop influenced the establishment of pathogens over there and a relatively higher *Lactobacillus* count along with a lower pH could decrease the occurrence of *Salmonella* in the crop [14]. It has been reported that the antimicrobial activity of organic acids is restricted within the crop and gizzard and any drastic changes of pH distally in the small intestine due to supplementation of organic acid are unlikely [3]. During the present investigation also supplementation of OAB decreased pH of only the gizzard, which corroborated the above hypothesis. It is intriguing to note that irrespective of dietary treatments pH in the gizzard was lower than that in the proventriculus, although the difference was not significant statistically. It may be mentioned that the pH mentioned in this article refers to that of the gut contents and not the pH of gut segments per se. The birds were slaughtered 2 hours postprandial and it is possible that post feeding acid production in the proventriculus passed off the peak level and the contents present there came down to the gizzard by the time the contents were collected. As a consequence, the contents present in the gizzard might show a comparatively lower pH than those of the proventriculus. Further, although there was little change in the population of *E. coli* and other coliforms, the lactobacilli increased in the OAB 1 dietary group. This was indicative of an antimicrobial action of the OAB at the upper alimentary tract. As it was discussed above, the antimicrobial action of OAB in the gizzard might have had reduced the number of the *Enterobacterioceae* and increased that of the *Lactobacillus* in the small intestine as a compensatory mechanism. Øverland et al. [12] reported that in growing-finishing pigs, population of the coliforms in the duodenum, jejunum, and ileum decreased when 1.2% potassium diformate was added to the diet. Gedek et al. [15] also reported that addition of 1.8% formic acid in diet reduced *E. coli* in the cecum and faeces. The coliforms in the present study can be assumed to be the representative for most of the enteric forms such as the *Salmonella*, *Shigella* and *Enterobacter* [16] and their reduction along with the *E. coli* wich ought to be beneficial for the host [12]. Because the microbial population is reduced, the metabolic needs are also reduced thereby increasing the availability of nutrients to the host. Supplemental OAB facilitated this process and thus augmented the performance of the experimental birds. On the other hand, a lower population of the enteric bacteria including the *Lactobacillus* in the BMD supplemented groups corroborated the earlier findings [17–19]. Decuypere et al. [20] reported that AGPs such as zinc bacitracin and virginiamycin may decrease the *Lactobacillus* and increase the coliforms in the alimentary tract of young pigs. In the present investigation also, the reduction in the number of *Lactobacillus* relative to that of the *E. coli* and the other coliforms was more in the BMD supplemented birds. 

Apart from pH and microbiological profile of the gut, histology of the small intestine might influence the performance of the birds in this study. Nutrient absorption in gut occurs from the intestinal mucosa and hence, manipulation thereon may improve the nutrient utilization *in vivo* [21–23]. In this study supplemental OAB increased the height of villi which probably facilitated nutrient absorption to a greater extent than that occurred in the BMD supplemented dietary group. Supplementation of probiotics in gut reportedly augmented growth and stability of specific bacteria which produce organic acids and this, in turn, might increase the height of the villi [22, 24]. It may be mentioned that the villus height in the BMD 1 group of birds was higher than the rest of the treatment groups in the jejunum where maximum digestion and absorption takes place because of a large luminal site and presence of more mature enterocytes [25]. This is quite intriguing since the performance of this particular group was comparatively inferior across the dietary treatments. However, it is worth mentioning that the villi in the jejunum of the birds receiving OAB as the dietary supplement were arranged in a zigzag fashion resembling a wave. According to Yamauchi and Isshiki [26], nutrient absorption occurs more efficiently when the villi are arranged in the aforementioned pattern than when they are positioned parallel. A wave like disposition of the villi would facilitate a better contact between the nutrients and the absorptive surface of the intestinal epithelium because the passage of feed through the alimentary canal becomes more time consuming through the zigzag flask than that occurring through a straight flask. This invariably results in a better nutrient utilization. The higher villus height coupled with a higher *Lactobacillus* count plausibly caused a better body weight and FCR in the OAB dietary groups.

It was concluded that dietary supplementation of organic acids may effectively replace AGPs as a growth promoter in broiler diets. Organic acids may yield superior effects in terms of growth performance compared to the AGPs such as BMD. The effects of organic acids may be related to a reduction in pH in gizzard and selective promotion of beneficial bacteria like *Lactobacillus* in the gut. Maintenance of villi in the small intestine at a greater height may be the other reason which makes the organic acids superior to the AGPs. Although, benefits of exceeding the dose of supplemental organic acids more than 1g/kg diet are not always conspicuous, based on the live weight and feed conversion data, supplementation of 2 g organic acid per kg diet may be recommended for total replacement of AGPs in broiler diet.

## Figures and Tables

**Table 1:** Composition of basal diet (g/kg unless stated otherwise).

Ingredients	Starter (d 1–21)	Finisher (d 22–35)
Ground maize	545	610
Soybean oil	—	7
De-oiled rice bran	35	46.8
Soybean meal (44% CP)	386	304
Oyster shell grit	18	17
Di-calcium phosphate	10	9
Common salt	1	1
Methionine	1.8	1.8
Lysine	0.5	0.7
NSP enzyme	0.5	0.5
Phytase	0.2	0.2
Vitamin and trace mineral premix^†^	2	2
Nutrient value (calculated value)		
Metabolizable energy MJ/kg	11.7	12.2
Crude protein	229	197
Calcium	9.5	8.7
Phosphorus	3.5	3.2
Lysine	3.4	4.15
Methionine	5.5	5.7

^†^Contained (per kg) retinyl acetate (3.75 mg), 1,25-hydroxycholecalciferol 4 mg, DL *α*-tocopheryl acetate 30 mg, menadione 4 mg, thiamine disulfide 3 mg, riboflavin tetrabutyrate 8 mg, methylcobalamine 0.025 mg, sodium pantothenate 15 mg, pyridoxine 5 mg, niacin 60 mg, biotin 0.2 mg, folic acid 2 mg and manganese 90 g, zinc 80 g, iron 90 g, copper 15 g (all as sulfate salt), iodine (as potassium iodide) 2 g, selenium (as sodium selenite) 0.3 g.

**Table 2:** Chemical composition (g/kg) and microbiological count (log_10_ CFU/g) of the experimental diets supplemented with an organic acid blend (OAB) and bacitracin methylene disalicylate (BMD).

	Diet	Dietary treatments					Significance (*P* value)		
		Control	OAB		BMD		Dose effect		Between treatments
Chemical compositin			1 g/kg diet	2 g/kg diet	0.5 g/kg diet	1.0 g/kg diet	OAB	BMD	
* *Crude protein*	Starter	226.6	228.7	230.1	229.2	228.7	NS	NS	NS
	Finisher	197.1	197.7	197.3	198.2	198.8	NS	NS	NS
* *Crud fiber	Starter	43.9	42.5	42.9	43.2	43.8	NS	NS	NS
	Finisher	37.6	35.4	35.9	36.8	37.2	NS	NS	NS
* *Ether extract	Starter	29.5	29.3	29.1	29.2	9.3	NS	NS	NS
	Finisher	38.7	38.8	38.6	38.5	8.7	NS	NS	NS
Microbial counts^†^									
Total coliforms	Starter	18	16	14	13	15	*	*	NS
	Finisher	19	12	14	16	11	*	*	NS
*Escherichia coli*	Starter	4	2	—	2	2	*	NS	*
	Finisher	4	3	—	2	1	*	*	NS
*Clostridium spp.*	Starter	210	180	170	200	180	*	*	*
	Finisher	200	180	160	180	160	*	*	*
*Aspergillus flavus* ^$^	Starter	—	—^‡^	2	1	—			
	Finisher	1	—^‡^	—	3	—			
*Aspergillus fumigatus* ^$^	Starter	2	—	—^‡^	—	—			
	Finisher	2	—	—	—	—			

^$^ Statistical analysis not performed due to lack of sufficient data; ^†^ *Salmonella *was negative for all the diets; ^‡^ mucoid growths were observed.

* Significance at *P* < .05; NS, not significant.

**Table 3:** Effects of feeding an organic acid blend (OAB) and bacitracin methylene disalicylate (BMD) at different dose levels on performance traits and carcass characteristics in broiler chicken ^†^.

	Dietary treatments					Pooled SE	Contrast *P* value		
Measurements	Control (Basal diet)	OAB		BMD			Dose effect		Between treatments
		1 g/kg diet	2 g/kg diet	0.5 g/kg diet	1.0 g/kg diet		OA	BMD	
Live performance and gross carcass traits									
Final live weight g	1510	1686	1773	1740	1717	13.2	***L	**LQ	***
Total gain g	1471	1648	1735	1702	1678	13.1	***L	** LQ	***
Overall FCR ^‡^	2.03	1.69	1.73	1.79	1.85	0.02	***LQ	**LQ	***
Dressing percentage	56.9	58.7	57.9	57.1	59.6	0.26	NS	**L	*
Breast g	220	290	261	283	310	9.3	*L	*L	NS
Thigh g	123	152	144	153	167	3.9	^$^	*L	NS
Drumstick g	125	151	146	166	187	4.4	NS	**L	*

Chemical composition (g/100 g meat on fresh basis) and nutrient accretion (g)									
Moisture	72.2	73.9	73.8	73.6	72.8	0.31	NS	NS	NS
Ash	3.59	6.89	5.41	3.66	3.92	0.15	**LQ	NS	**
Protein	21.9	22.1	22.1	20.4	21.8	0.21	NS	^$^	NS
Protein accretion	321.8	384.9	383.9	347.6	366.4	4.37	**L	^$^	***
Ash accretion	50.2	113.4	92.5	60.2	63.7	2.54	**LQ	NS	***

^†^The birds were on test for 35 days, ^‡^Feed conversion ratio, calculated by dividing total feed intake by total live weight gain in 35 days. ^L^significant linear effect, ^Q^significant quadratic effect, *at *P* < .05, **at *P* < .01, ***at *P* < .001, ^$^ *P* < .1.

**Table 4:** Effect of feeding organic acid blend (OA) and bacitracin methylene disalicylate (BMD) at different dose levels on pH of different segments of the gastrointestinal tract, microbial counts in the digesta contents of the small intestine and length of the villi in different segments of the small intestine^ †^.

	Dietary treatments					Pooled SE	Contrast *P* value		
Measurements	Control (Basal diet)	OAB		BMD			Dose effect		Between treatments
		1 g/kg diet	2 g/kg diet	0.5 g/kg diet	1.0 g/kg diet		OA	BMD	
pH in different segments of the gastrointestinal tract									
Crop	4.75	4.90	4.71	4.78	5.32	0.069	NS	NS	NS
Proventriculus	3.03	2.95	3.07	3.13	2.4	0.089	NS	^$^	NS
Gizzard	3.23	2.63	2.61	3.15	2.52	0.059	**LQ	**L	NS
Duodenum	6.18	6.12	6.27	5.98	6.38	0.06	NS	NS	NS
Jejunum	6.32	6.29	6.28	6.35	6.34	.09	NS	NS	NS
Ileum	6.97	7.17	7.18	7.4	7.28	0.055	NS	^$^	NS

Microbial counts of the digesta in different segments of the small intestine (log_10_ CFU/g digesta)									
*Escherichia coli *	3.78	3.38	3.7	2.22	1.27	0.582	NS	NS	NS
Other coliforms	2.72	2.6	2.63	2.65	0.63	0.422	NS	NS	NS
*Lactobacillus *spp.	2.43	6.5	1.5	1.5	1.2	0.32	**Q	NS	*

Height of villi (*μ*m) in different segments of small intestine									
Duodenum	1513	1524	1667	1197	1238	25.74	*L	**LQ	***
Jejunum	996	1049	1063	984	1287	16.37	*Q	***LQ	*
Ileum	799	752	822	697	747	7.46	*LQ	***LQ	*

^†^Birds were harvested on d 35,  ^L^significant linear effect, ^Q^significant quadratic effect, *at *P* < .05, **at *P* < .01, *at ^$^ *P* < .001, *P* < .1.

Tests for *Salmonella* and *Clostridium* were negative and hence the results are not shown.
